# Supplementary material for: The microbiome biomarkers of pregnant women’s vaginal area predict preterm prelabor rupture in Western China
Source: Front Cell Infect Microbiol. 2024 Oct 31;14:1471027. doi: 10.3389/fcimb.2024.1471027 (PMC11560878; doi:10.3389/fcimb.2024.1471027)
Supplement: Supplementary file 1 [file DataSheet1.zip › compare_1/Community/Pieplot/species/group/P.species.pieplot.pdf]

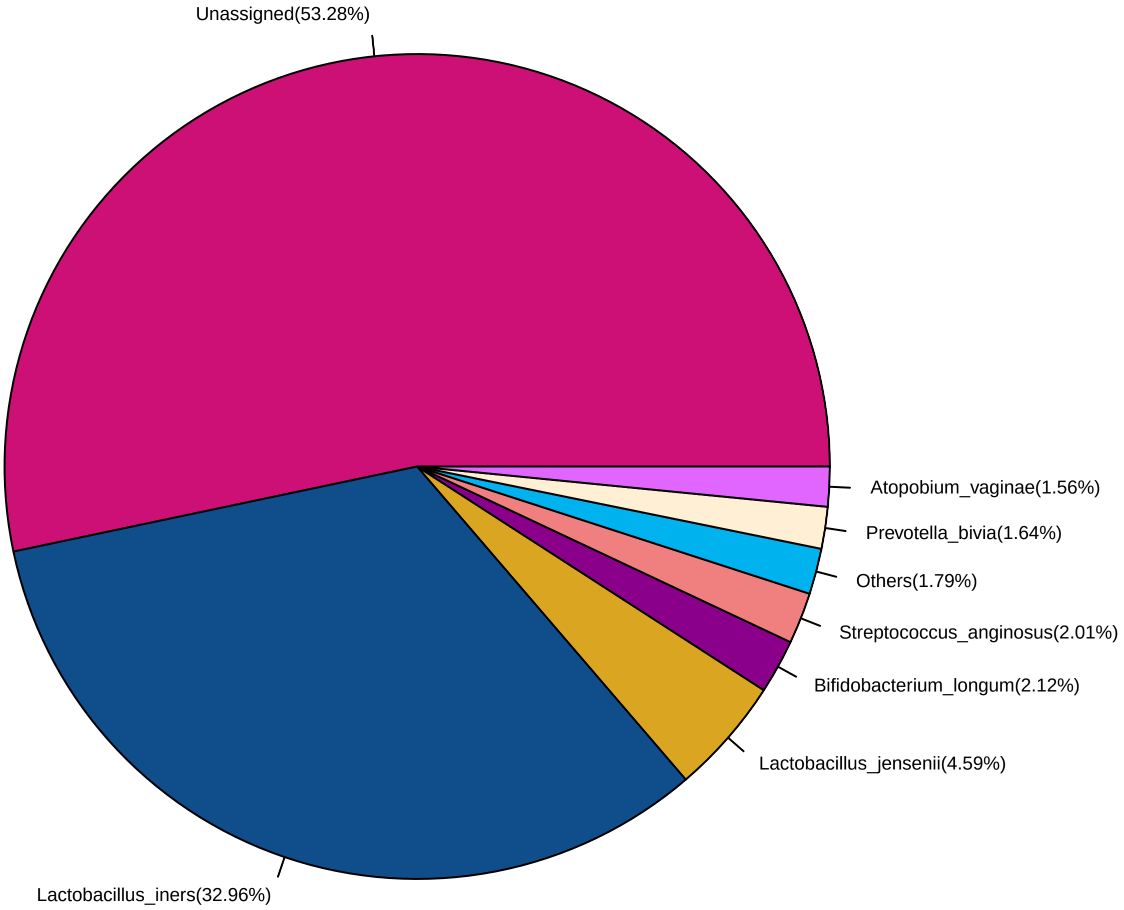

Unassigned(53.28%)
Atopobium_vaginae(1.56%)
Prevotella_bivia(1.64%)
Others(1.79%)
Streptococcus_anginosus(2.01%)
Bifidobacterium_longum(2.12%)
Lactobacillus_jensenii(4.59%)
Lactobacillus_iners(32.96%)
